# Supplementary material for: A 4D transcriptomic map for the evolution of multiple sclerosis-like lesions in the marmoset brain
Source: bioRxiv. 2023 Sep 27:2023.09.25.559371. Preprint. [Version 1] doi: 10.1101/2023.09.25.559371 (PMC10557631; doi:10.1101/2023.09.25.559371)
Supplement: 1 [file NIHPP2023.09.25.559371V1-supplement-1.pdf]

bioRxiv preprint doi: https://doi.org/10.1101/2023.09.25.559371; this version posted September 27, 2023. The copyright holder for this preprint (which was not certified by peer review) is the author/funder. This article is a US Government work. It is not subject to copyright under 17 USC 105 and is also made available for use under a CC0 license.

Fig S1 - related to Fig1

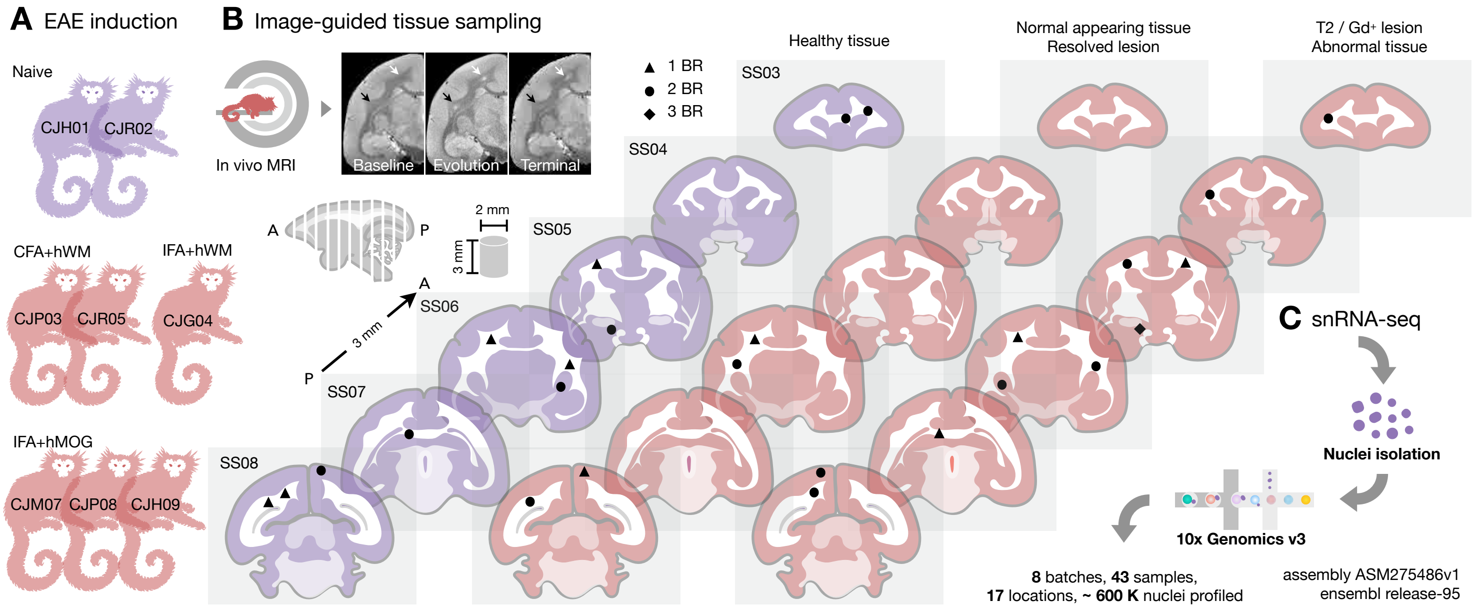

D Sample index

Coarse	Fine	He.Control		NA.Control		Re.Lesion		T2.Lesion		Gd.Lesion		Ab.Tissue	
"WM"	fWM	2	CJH01, CJR02					3	CJP03, CJG04, CJP08	1	CJM07		
	tWM	2	CJH01, CJR02	1	CJM07	3	CJP03, CJR05 x2	1	CJH09	1	CJM07		
	pWM	3	CJH01 x2, CJR02	1	CJG04			6	CJP03 x2, CJM07 x2, CJP08, CJH09				
	aCC	2	CJH01, CJR02										
	pCC	2	CJH01, CJR02					1	CJP08				
	OpT	2	CJH01, CJR02					3	CJM07, CJP08, CJH09				
"other"	LGN	2	CJH01, CJR02									2	CJP08, CJH09
"GM"	pCTX	2	CJH01, CJR02	1	CJH09			2	CJP08, CJH09				

bioRxiv preprint doi: https://doi.org/10.1101/2023.09.25.559371; this version posted September 27, 2023. The copyright holder for this preprint (which was not certified by peer review) is the author/funder. This article is a US Government work. It is not subject to copyright under 17 USC 105 and is also made available for use under a CC0 license.

FigS1. related to Fig1. Experimental design used to create a transcriptome map for the evolution of white matter (WM) lesions with single nucleus resolution.

(A) The dataset includes marmosets that were inoculated with human white matter (hWM) or recombinant human myelin oligodendrocyte glycoprotein (hMOG) emulsified in complete (CFA) or incomplete Freund's adjuvant (IFA) to induce experimental autoimmune encephalomyelitis (EAE) and healthy, naïve controls.
(B) The experimental workflow involved scanning and categorizing brain tissue using MRI. Postmortem brains were sliced into 3-mm slabs from anterior (A) to posterior (P). Specific areas of interest were sampled as cylinders with a diameter of 2 mm and height of 3 mm. These sampled areas were labeled on the standard slab (SS) index and grouped based on disease condition. The number of biological repeats (BR) is indicated by black annotations on the SS.
(C) Nuclei were isolated from the sampled areas to prepare cDNA libraries, which were then sequenced.
(D) Sampled areas were categorized into 3 types: coarse brain region, fine tissue location, and disease condition. Abbreviations: f (frontal), t (temporal), p (parietal), WM (white matter), a (anterior), p (posterior), CC (corpus callosum), OpT (optic tract), CTX (cortex), LGN (lateral geniculate nucleus), EAE (experimental autoimmune encephalomyelitis), He (healthy), NA (normal-appearing), Re (resolved), T2 (T_2-hyperintense MRI detected), Gd (gadolinium), and Ab (abnormal).

bioRxiv preprint doi: https://doi.org/10.1101/2023.09.25.559371; this version posted September 27, 2023. The copyright holder for this preprint (which was not certified by peer review) is the author/funder. This article is a US Government work. It is not subject to copyright under 17 USC 105 and is also made available for use under a CC0 license.

Fig S2 - related to Fig1

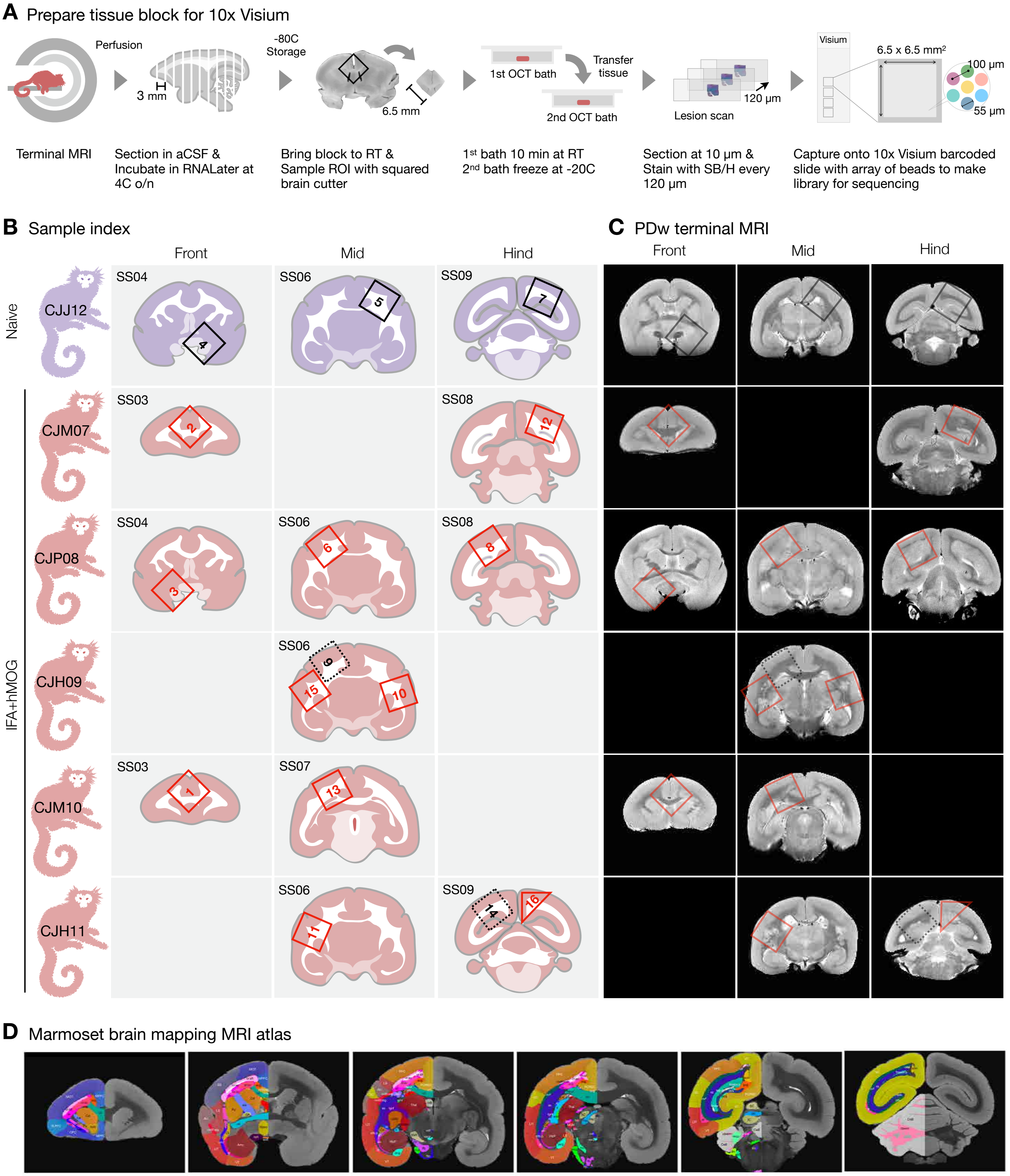

bioRxiv preprint doi: https://doi.org/10.1101/2023.09.25.559371; this version posted September 27, 2023. The copyright holder for this preprint (which was not certified by peer review) is the author/funder. This article is a US Government work. It is not subject to copyright under 17 USC 105 and is also made available for use under a CC0 license.

FigS2. related to Fig1. Experimental design for creating a transcriptome map of white matter (WM) lesions with spatial resolution.

(A) The experimental workflow involves identifying WM lesions using magnetic resonance imaging (MRI) and preparing postmortem tissue for spatial transcriptome analysis using the 10x Visium platform. Abbreviations: aCSF (artificial cerebrospinal fluid), RT (room temperature), ROI (region of interest), OCT (optimal cutting temperature), SB (Sudan black), H (hematoxylin).

(B) The dataset includes marmosets inoculated with recombinant human myelin oligodendrocyte glycoprotein (hMOG) emulsified in incomplete Freund's adjuvant (IFA). Sampled areas were blocked (6.5x6.5x3 mm^3), labeled onto the standard slab (SS) index, and grouped by matching brain area across diseased conditions.

(C) Proton density-weighted (PDw) MRI was performed at the terminal time point and matched with the sampled tissue areas.

(D) A postmortem MRI atlas overlaid with brain region labels was used to match with the selected terminal PDw MRI.

bioRxiv preprint doi: https://doi.org/10.1101/2023.09.25.559371; this version posted September 27, 2023. The copyright holder for this preprint (which was not certified by peer review) is the author/funder. This article is a US Government work. It is not subject to copyright under 17 USC 105 and is also made available for use under a CC0 license.

Fig S3 - related to Fig1

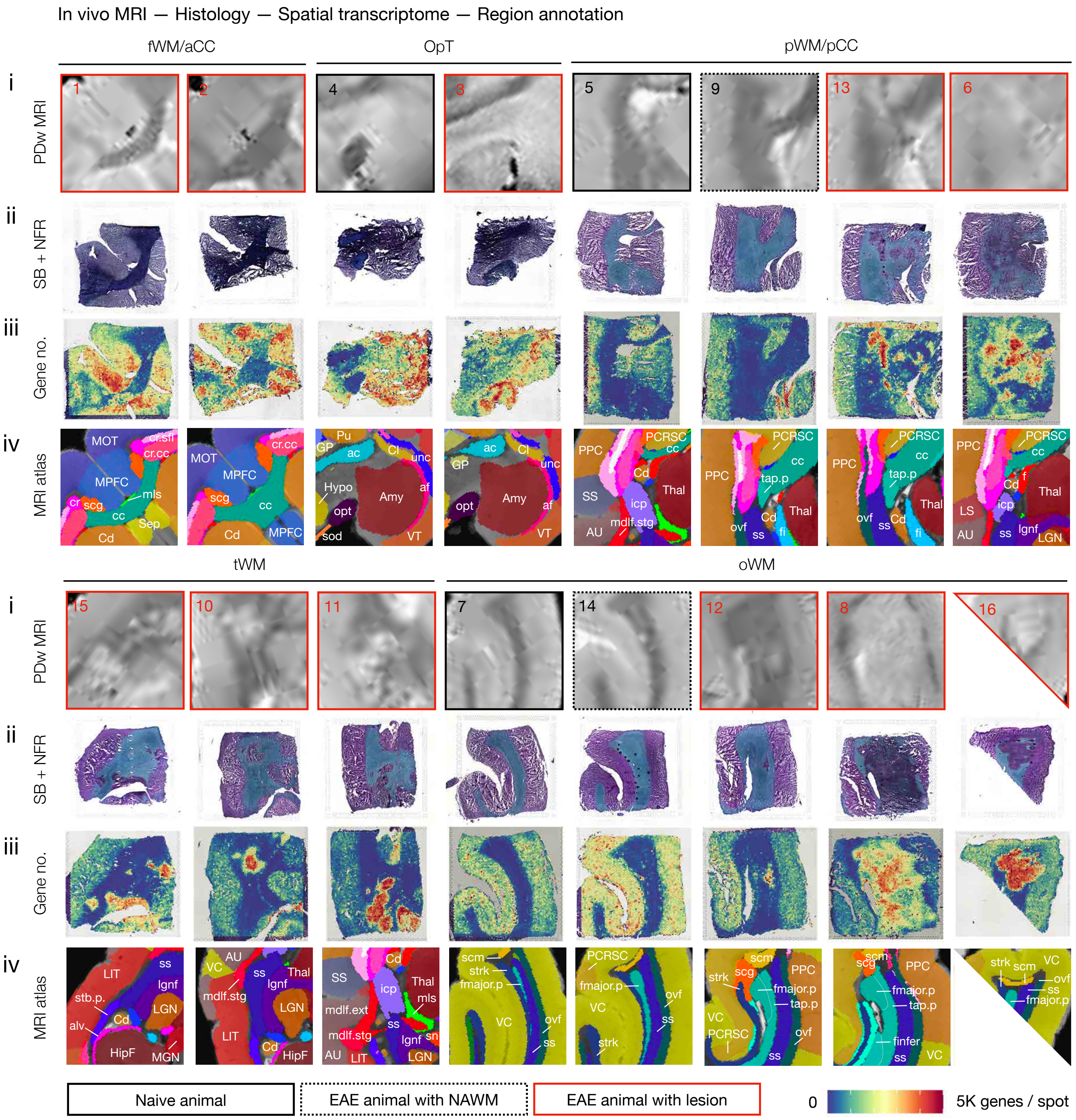

bioRxiv preprint doi: https://doi.org/10.1101/2023.09.25.559371; this version posted September 27, 2023. The copyright holder for this preprint (which was not certified by peer review) is the author/funder. This article is a US Government work. It is not subject to copyright under 17 USC 105 and is also made available for use under a CC0 license.

FigS3. related to Fig1. Matched brain regions imaged by different modalities.

i) In vivo brain PDw MRI acquired on a 7 Tesla scanner at the disease terminal.
ii) Histological examination of myelin content using Sudan black (SB) and nuclear fast red (NFR) staining of postmortem tissue.
iii) RNA landscape visualized through the 10x Visium platform.
iv) Marmoset MRI atlas with region annotations.

See source data for the full list of abbreviations for brain regions.

bioRxiv preprint doi: https://doi.org/10.1101/2023.09.25.559371; this version posted September 27, 2023. The copyright holder for this preprint (which was not certified by peer review) is the author/funder. This article is a US Government work. It is not subject to copyright under 17 USC 105 and is also made available for use under a CC0 license.

Fig S4 - related to Fig1

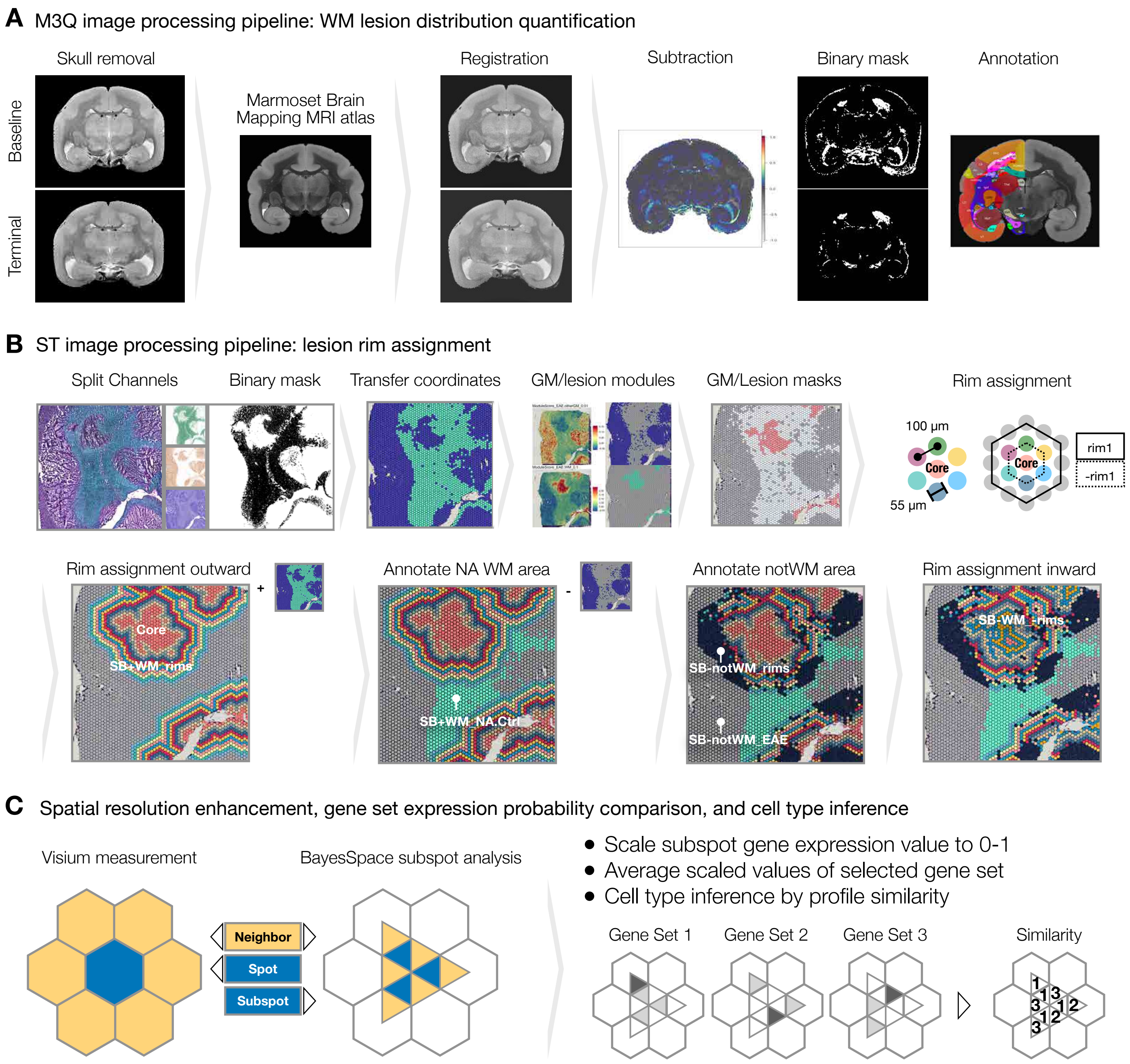

bioRxiv preprint doi: https://doi.org/10.1101/2023.09.25.559371; this version posted September 27, 2023. The copyright holder for this preprint (which was not certified by peer review) is the author/funder. This article is a US Government work. It is not subject to copyright under 17 USC 105 and is also made available for use under a CC0 license.

FigS4. related to Fig1. Image processing and resolution enhancement pipelines used to quantify white matter (WM) lesion load, generate lesion subregion masks, and deconvolute mixed transcript signals.

(A) To gain insight into lesion development and progression, an MRI characterization of MS-like lesions in the Marmoset Quantitatively (M3Q) pipeline was developed, including the following steps (**Methods**): Proton density-weighted (PDw) MRI images at baseline (before EAE induction) and terminal (before tissue collection) time points were subjected to the N4 bias field correction algorithm. The brain portion of the images, corresponding to the region of interest, was extracted using a skull-removal algorithm to improve image alignment. Each image was individually registered to the marmoset MRI atlas using bUnwarpJ, achieving spatial alignment across time points and animals. MRI intensity changes were calculated by subtracting the normalized terminal image from the baseline image, providing information about lesion location. Binary lesion masks were created by applying intensity thresholding to the subtracted images, segmenting the region of interest. The WM portion of the lesion mask was parsed and analyzed using atlas annotation indexing, enabling further characterization of the lesions based on location and distribution.

(B) To gain insight into the regionally enriched signal distribution within and near the WM lesion, a spatial transcriptome (ST) image processing pipeline was employed with the following steps: The myelinated WM area (Sudan black-positive) was extracted using the "Color Deconvolution" function in Fiji with the default "H DAB" setting, resulting in an SB$^+$ WM binary mask. The coordinates of the SB$^+$ WM mask were transferred to the 10x Visium spot hexagon coordinate system using Seurat, facilitating the spatial mapping of gene expression data. To distinguish SB$^-$ gray matter (GM) from SB$^-$ demyelinated WM, GM and lesion gene module scores were calculated and filtered to create GM and lesion masks accordingly. Spots that exhibited both SB$^-$ and IMM$^+$ signals were identified as the lesion core. Next, 10 concentric rims (SB$^+$WM_rims) extending outward from the lesion core were assigned to mark the adjacent lesion neighborhoods. The normal-appearing (NA) WM area was annotated by subtracting the lesion neighborhoods from the SB$^+$ WM mask in animals with experimental autoimmune encephalomyelitis (EAE), and this region was labeled as "SB$^+$WM_NA.Ctrl." Additionally, lesion neighborhoods that overlapped with the GM mask were labeled as "SB$^-$notWM_rims," while the supplemental area was labeled as "SB$^-$notWM_EAE" in animals with EAE. Subregions within the lesion core were further divided based on centripetal rim assignments (SB$^-$WM_-rims). For healthy animals, "SB$^+$WM_He.Ctrl" and "SB$^-$notWM_He" labels were used to annotate tissue with or without SB staining, respectively.

(C) The spatial transcriptome resolution, initially measured at the spot level using the 10x Visium platform, was further enhanced to the subspot level using the BayesSpace algorithm. The enhanced signals at the subspot level were then rescaled to a ratio of 1 across genes. To gain insights into cell-type distributions, the averaged expression of gene sets enriched in specific cell types, acquired from single-nucleus RNA sequencing (snRNA-seq) references, was calculated. Cell-type locations were inferred by assessing the relative profile similarity score, allowing identification and mapping of different cell types within the spatial transcriptome data. By employing these techniques, the spatial transcriptome analysis achieved higher resolution, enabling a more detailed understanding of the cellular composition and gene expression patterns within the tissue of interest.

bioRxiv preprint doi: https://doi.org/10.1101/2023.09.25.559371; this version posted September 27, 2023. The copyright holder for this preprint (which was not certified by peer review) is the author/funder. This article is a US Government work. It is not subject to copyright under 17 USC 105 and is also made available for use under a CC0 license.

Fig S5 - related to Fig2

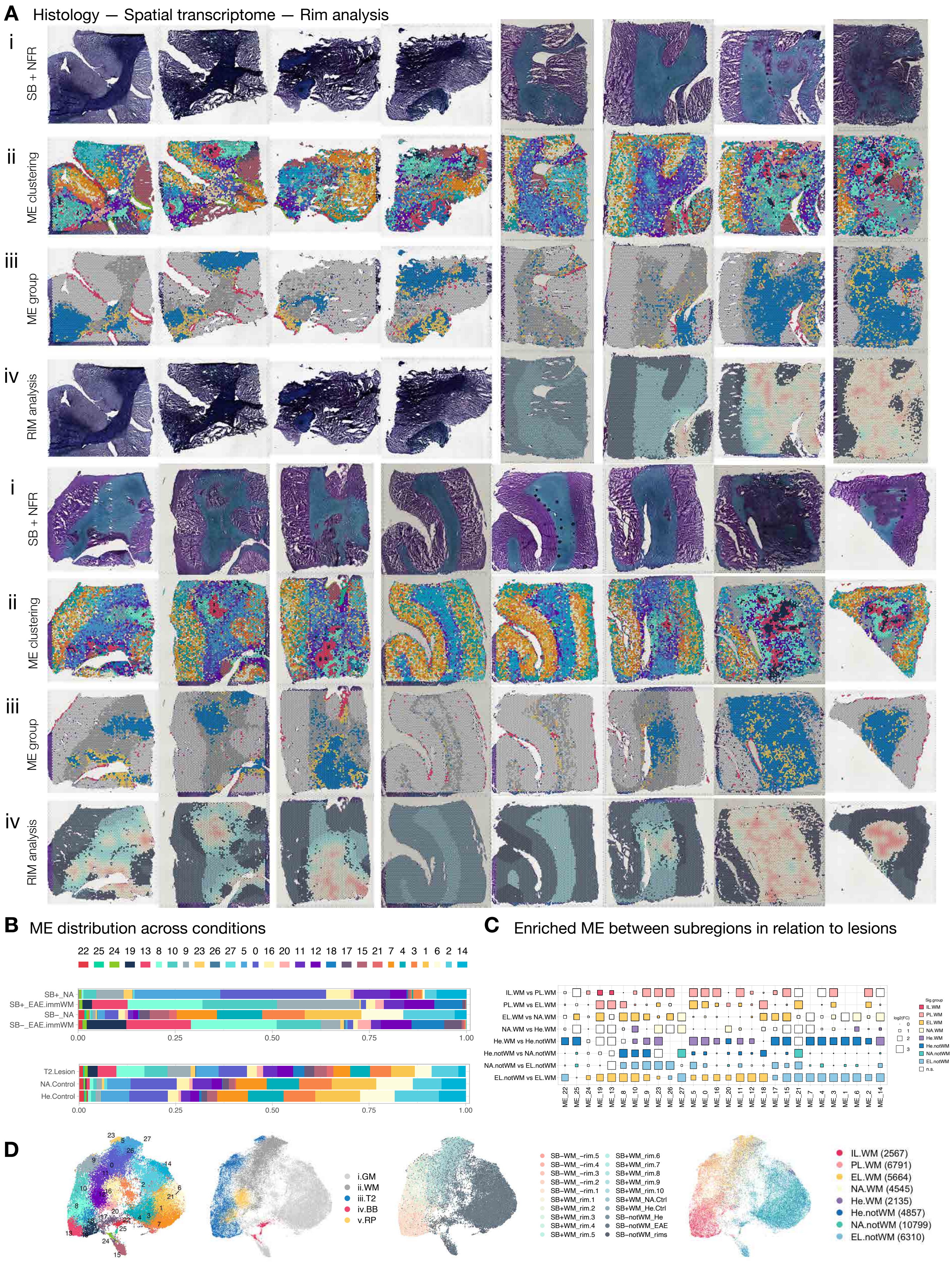

bioRxiv preprint doi: https://doi.org/10.1101/2023.09.25.559371; this version posted September 27, 2023. The copyright holder for this preprint (which was not certified by peer review) is the author/funder. This article is a US Government work. It is not subject to copyright under 17 USC 105 and is also made available for use under a CC0 license.

FigS5. related to Fig2. Matched brain regions of interest across pathological states are color-coded based on different microenvironment (ME) phenotypes.

(A) To demonstrate the phenotypic characterization of the ME within matched brain regions, contrasts between: i) myelin content visualized through Sudan black (SB) and nuclear fast red (NFR) staining, ii) unbiased ME clustering achieved through transcriptome similarity analysis, iii) 5 ME groups assigned by ME profile similarity, and iv) lesion subregions assigned using rim analysis and overlaid onto SB/NFR stained images, were indexed. The "Color Deconvolution" (**FigS4B**) for Samples 1–4 was unsuccessful due to suboptimal contrast between SB and NFR staining, resulting in their exclusion from the lesion subregion assignment in the rim analysis; however, they are included for ME clustering analysis.

(B) Stacked bar plots show the relative proportions of transcriptomic ME at the spot resolution across different pathological states (top), as well as the levels of myelin and inflammation (bottom).

(C) Dot plots depict the change in spot proportion across subregional white matter (WM) of Samples 5–16. Significantly (FDR < 0.05 & abs(Log2FC) > 0.5) enriched ME between pairs of subregional WM area are colored accordingly. “IL.WM” contains SB-WM_-rim5 to SB-WM_-rim2, “PL.WM” contains SB-WM_-rim1 to SB+WM_rim1, “EL.WM” contains SB+WM_rim2 to SB+WM_rim10), “NA.WM” contains SB+WM_NA.Ctrl, “He.WM” contains SB+WM_He.Ctrl, “He.notWM” contains SB-notWM_He, “NA.notWM” contains SB-notWM_EAE, and “EL.noWM” contains SB-notWM_rims.

(D) UMAP plots colored by ME cluster (**left**), subregions assigned by rim analysis (**middle**), and subregional WM (**right**).

bioRxiv preprint doi: https://doi.org/10.1101/2023.09.25.559371; this version posted September 27, 2023. The copyright holder for this preprint (which was not certified by peer review) is the author/funder. This article is a US Government work. It is not subject to copyright under 17 USC 105 and is also made available for use under a CC0 license.

Fig S6 - related to Fig2

A Spatial transcriptome ME clustering & subregional rim annotation

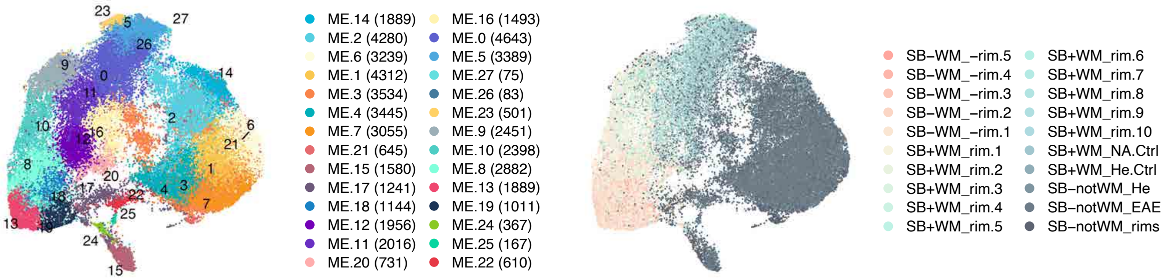

B Gene module & gene ontology (GO) analysis

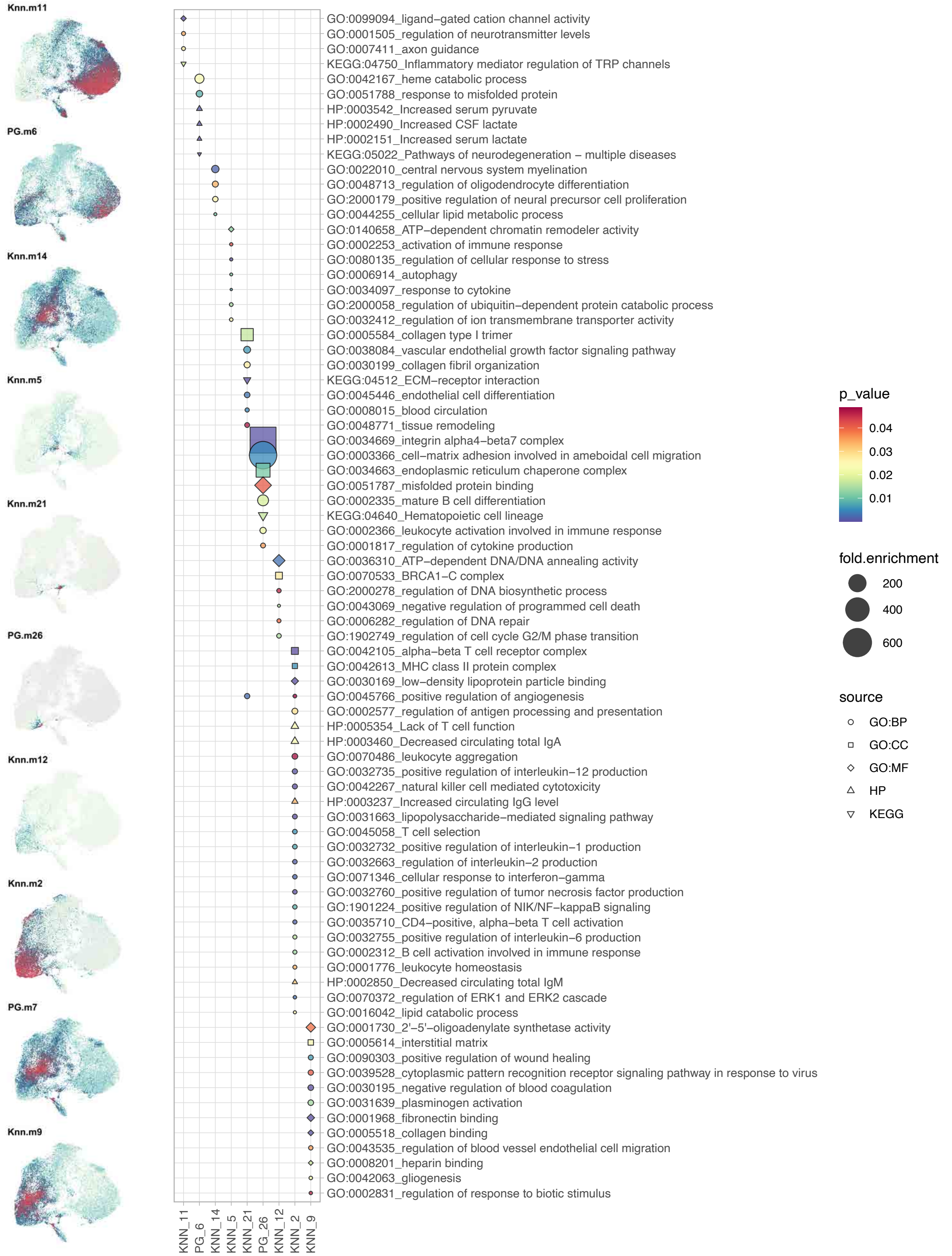

bioRxiv preprint doi: https://doi.org/10.1101/2023.09.25.559371; this version posted September 27, 2023. The copyright holder for this preprint (which was not certified by peer review) is the author/funder. This article is a US Government work. It is not subject to copyright under 17 USC 105 and is also made available for use under a CC0 license.

FigS6. related to Fig2. Spatial organization of transcriptomes within different microenvironments and functional enrichment of gene modules within subregions.

(A) UMAP scatter plots color-coded by microenvironment (ME) clustering and subregional labeling. The number of spots per microenvironment is indicated in parentheses. Abbreviations: SB (Sudan black), WM (white matter), NA (normal appearing), He (healthy), Ctrl (control).

(B) UMAP scatter plots color-coded by gene expression. Gene modules are annotated with enriched Gene Ontology (GO) terms, including three major subontologies: Molecular Functions (MF), Biological Process (BP), and Cellular Component (CC). When available, additional annotations from the KEGG and HP databases are included. Specifically, the Knn.m11 gene module is enriched in ME14, 2, 6, 1, 3, 4, 7, 21, 15, 17, which dominate the SB-notWM (gray matter) area and are involved in the regulation of neurotransmitter levels, as expected. Perilesional (SB+WM_rims) and lesional (SB-WM_rims) ME are enriched with modules involved in various processes, including myelination and lipid metabolism (Knn.m14), glycometabolism and neurodegeneration (PG.m6), immune and stress response (Knn.m5 and Knn.m2), extracellular matrix (ECM) and vascular function (Knn.m21 and Knn.m9), hematopoietic and leukocytic cell development (PG.m26 and Ken.m2), and programmed cell death and cell cycle (Knn.m12).

bioRxiv preprint doi: https://doi.org/10.1101/2023.09.25.559371; this version posted September 27, 2023. The copyright holder for this preprint (which was not certified by peer review) is the author/funder. This article is a US Government work. It is not subject to copyright under 17 USC 105 and is also made available for use under a CC0 license.

Fig S7 - related to Fig2

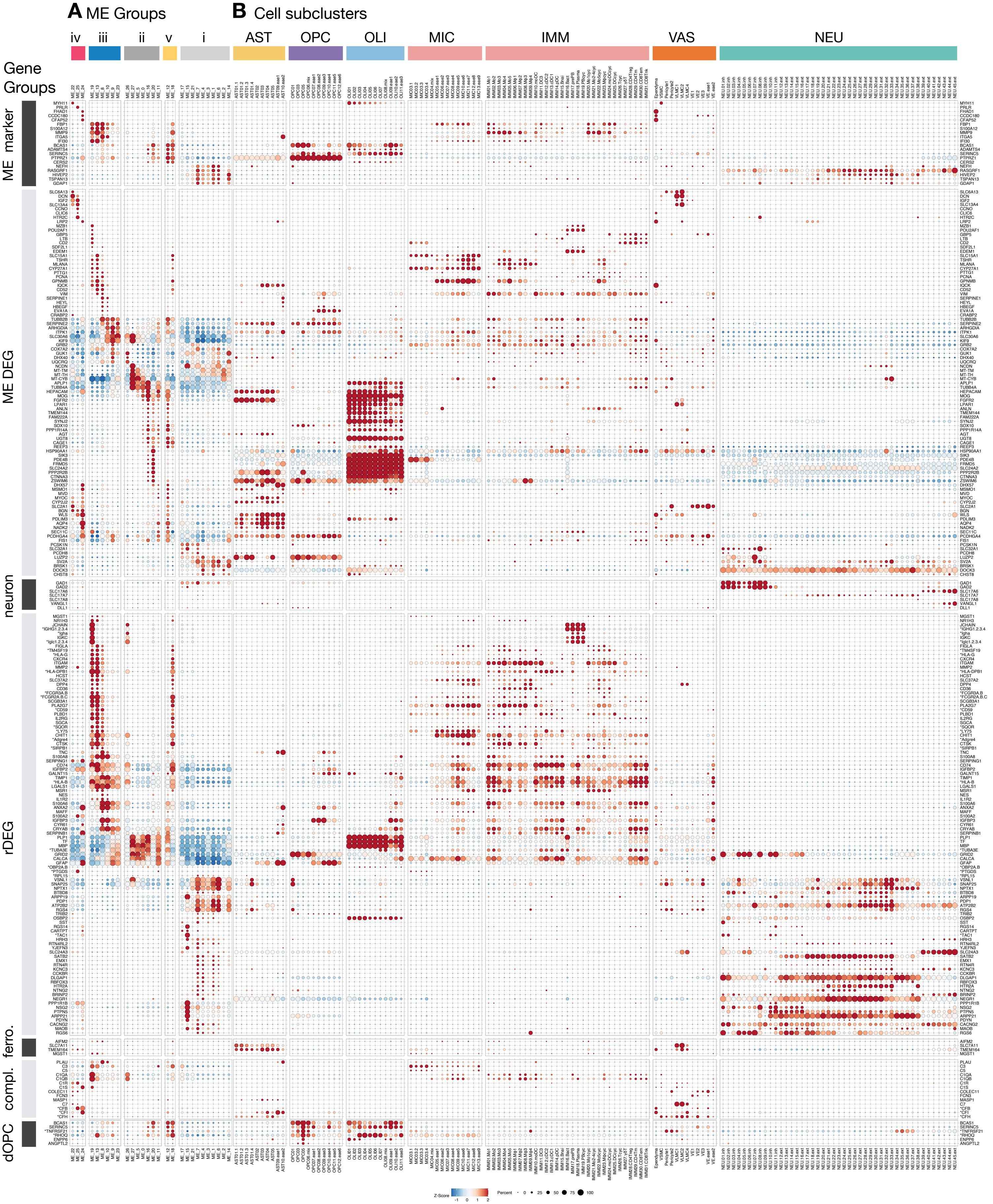

bioRxiv preprint doi: https://doi.org/10.1101/2023.09.25.559371; this version posted September 27, 2023. The copyright holder for this preprint (which was not certified by peer review) is the author/funder. This article is a US Government work. It is not subject to copyright under 17 USC 105 and is also made available for use under a CC0 license.

Fig S7.continued - related to Fig2

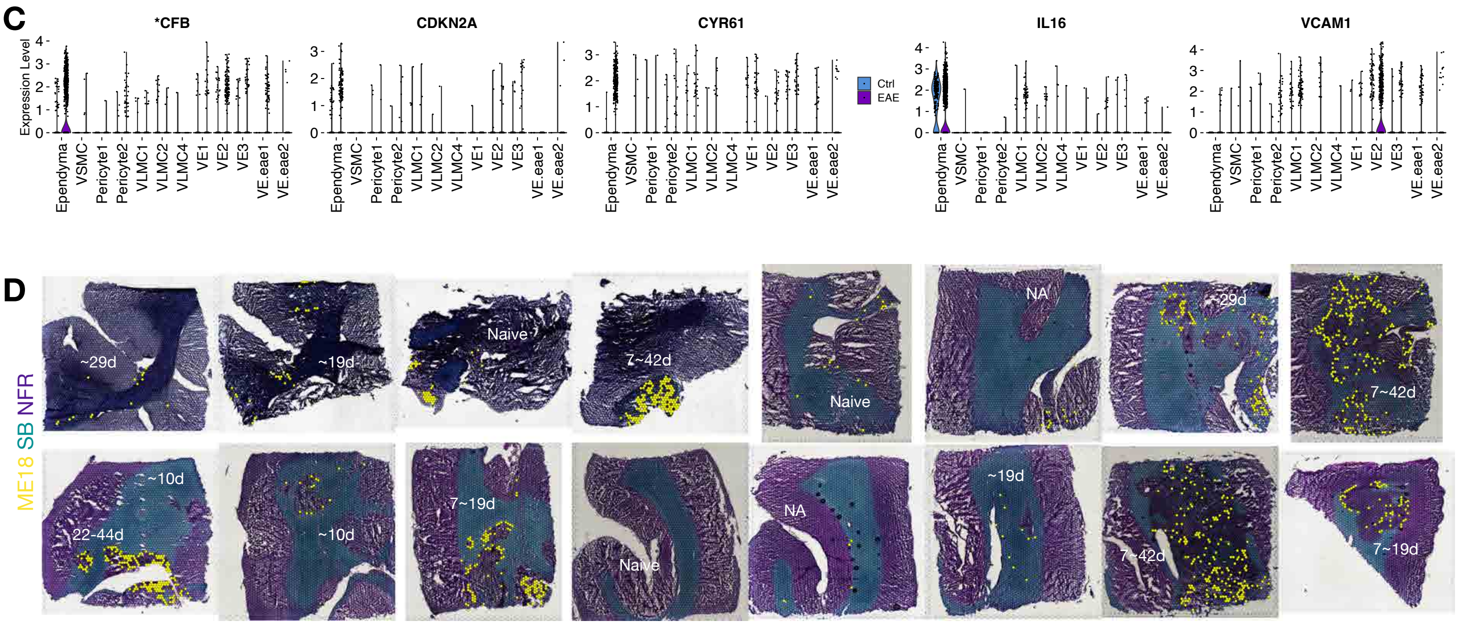

bioRxiv preprint doi: https://doi.org/10.1101/2023.09.25.559371; this version posted September 27, 2023. The copyright holder for this preprint (which was not certified by peer review) is the author/funder. This article is a US Government work. It is not subject to copyright under 17 USC 105 and is also made available for use under a CC0 license.

FigS7. related to Fig2. Complement factor B (CFB) expression is elevated in EAE ependyma compared to control, and ME18 is enriched in older lesions.

(A) Dot plot showing the averaged and scaled expression of selected genes across microenvironments (ME). Gene names starting with "*" indicate human (hs) or mouse (mm) orthologs of marmoset gene identification numbers (See **Table S9** for the full list).

(B) Dot plot showing the averaged and scaled expression of selected genes across L2 subclusters. Genes are split into groups to aid label tracking. Abbreviations: ME marker (genes used to annotate ME groups in **Fig2A**), ME DEG (differentially expressed genes across 28 ME), rDEG (regional differentially expressed genes), ferro. (ferroptosis genes), compl. (complement genes), dOPC (differentiating OPC enriched genes).

(C) Violin plot showing the expression of **CFB* (human homolog of marmoset ENSCJAG00000048204), *CDKN2A*, *CYR61*, *IL16*, and *VCAM1* across vascular cells in control and EAE.

(D) SB/NFR-stained tissue across 16 ROI labeled by the distribution of ME18 (yellow dots) and annotated by lesion age, dated by longitudinal MRI (**Methods**).

bioRxiv preprint doi: https://doi.org/10.1101/2023.09.25.559371; this version posted September 27, 2023. The copyright holder for this preprint (which was not certified by peer review) is the author/funder. This article is a US Government work. It is not subject to copyright under 17 USC 105 and is also made available for use under a CC0 license.

Fig S8 - related to Fig2

A L1 and L2 gene list used to infer cell types

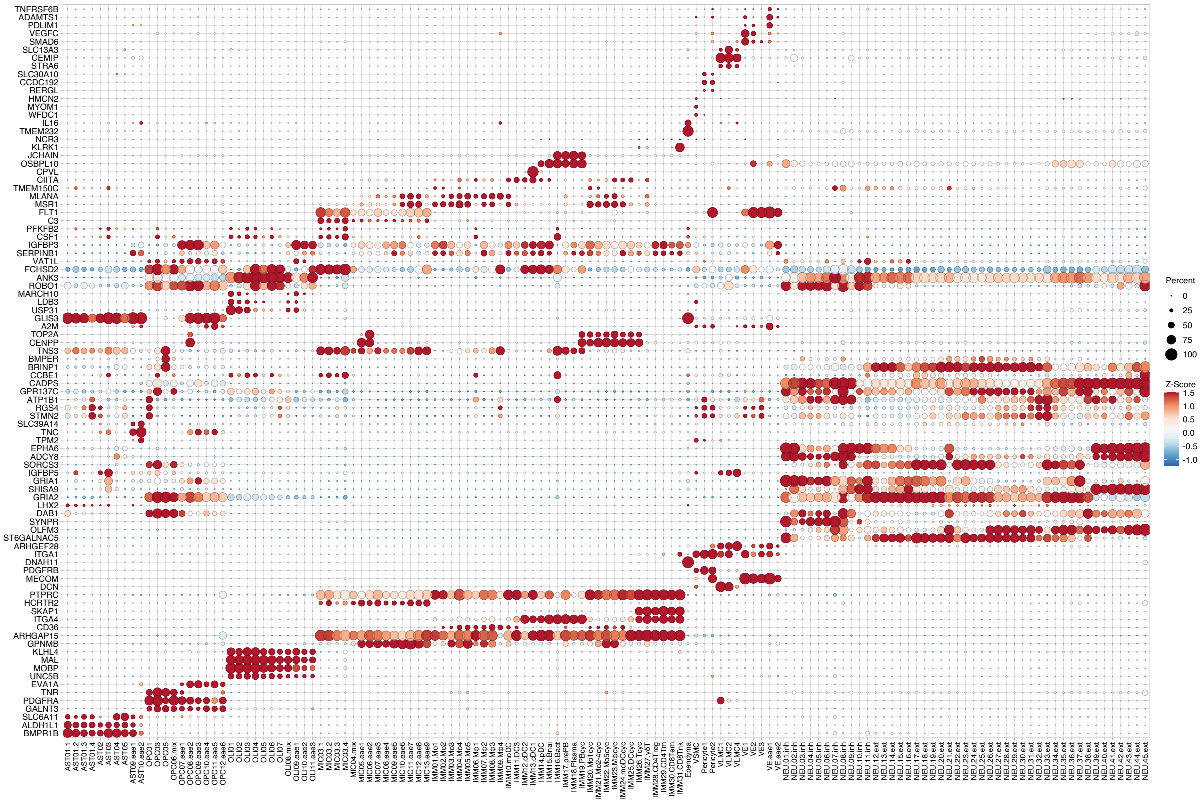

B Inferred cell type distribution across subspots of 16ROI and disease states

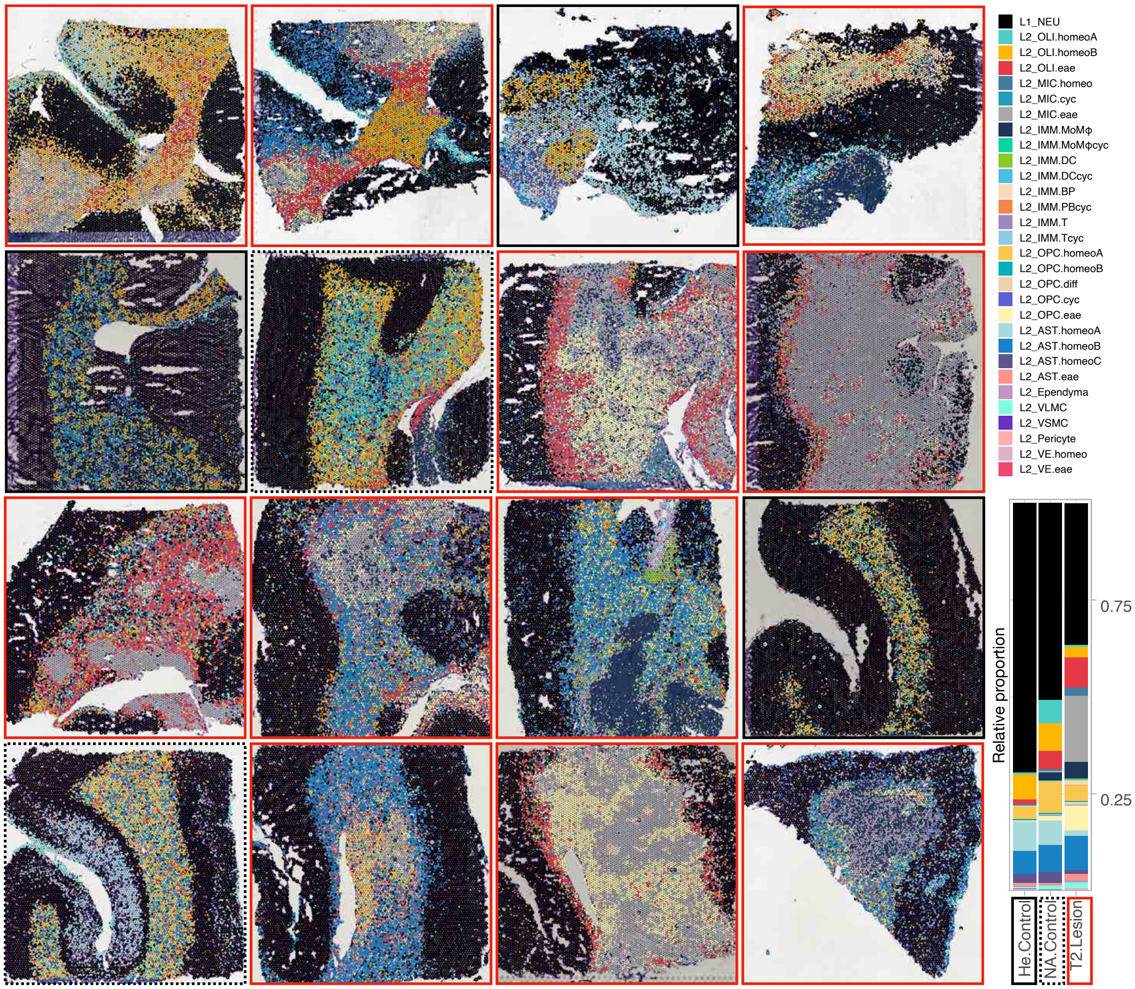

bioRxiv preprint doi: https://doi.org/10.1101/2023.09.25.559371; this version posted September 27, 2023. The copyright holder for this preprint (which was not certified by peer review) is the author/funder. This article is a US Government work. It is not subject to copyright under 17 USC 105 and is also made available for use under a CC0 license.

FigS8. related to Fig2. Selected L1 and L2 genes for each cell class and subcluster.

(A) Dot plot showing the averaged and scaled expression of selected genes used to infer cell types for BayesSpace-enhanced subspots across L2 subclusters.
(B) BayesSpace-enhanced subspots colored by inferred cell types across 16 ROI labeled by crude disease category.

bioRxiv preprint doi: https://doi.org/10.1101/2023.09.25.559371; this version posted September 27, 2023. The copyright holder for this preprint (which was not certified by peer review) is the author/funder. This article is a US Government work. It is not subject to copyright under 17 USC 105 and is also made available for use under a CC0 license.

Fig S9 - related to Fig3

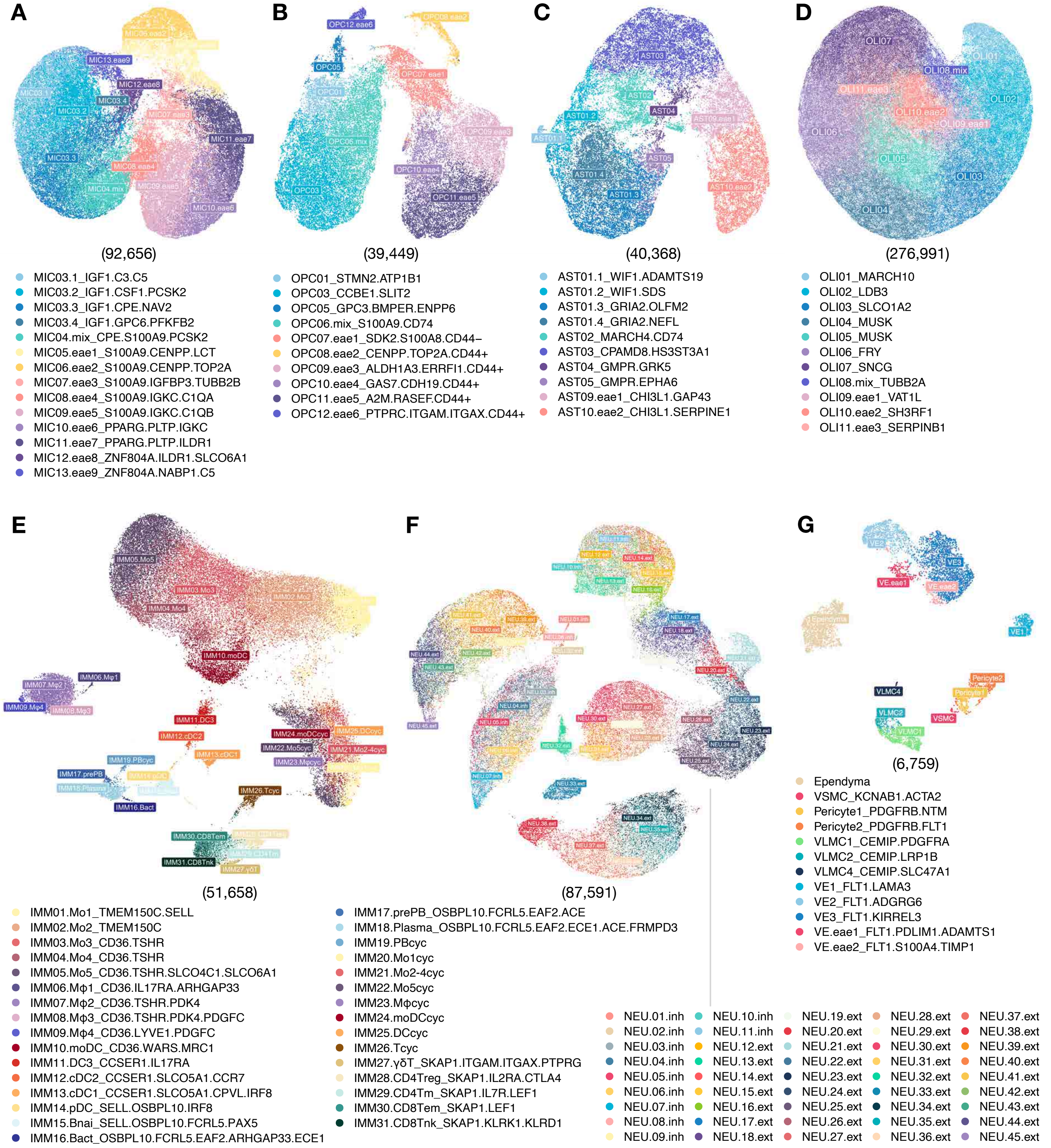

bioRxiv preprint doi: https://doi.org/10.1101/2023.09.25.559371; this version posted September 27, 2023. The copyright holder for this preprint (which was not certified by peer review) is the author/funder. This article is a US Government work. It is not subject to copyright under 17 USC 105 and is also made available for use under a CC0 license.

FigS9. related to Fig3. UMAP scatter plot of the level 2 analysis conducted on major cell classes.

(A) – (G) The level 1 (L1) analysis identified 6 major cell classes, which were further divided into 7 partitions in the level 2 (L2) analysis. UMAP scatter plots show subclustering of the following cell classes: microglia (MIC), oligodendrocyte progenitor cells (OPC), astrocytes (AST), oligodendrocytes (OLI), peripherally derived immune cells (P.IMM), neurons (NEU), and vascular/meningeal/ventricular cells (VAS). The number of nuclei analyzed in each L2 UMAP plot is listed in parentheses.

bioRxiv preprint doi: https://doi.org/10.1101/2023.09.25.559371; this version posted September 27, 2023. The copyright holder for this preprint (which was not certified by peer review) is the author/funder. This article is a US Government work. It is not subject to copyright under 17 USC 105 and is also made available for use under a CC0 license.

Fig S10 - related to Fig3

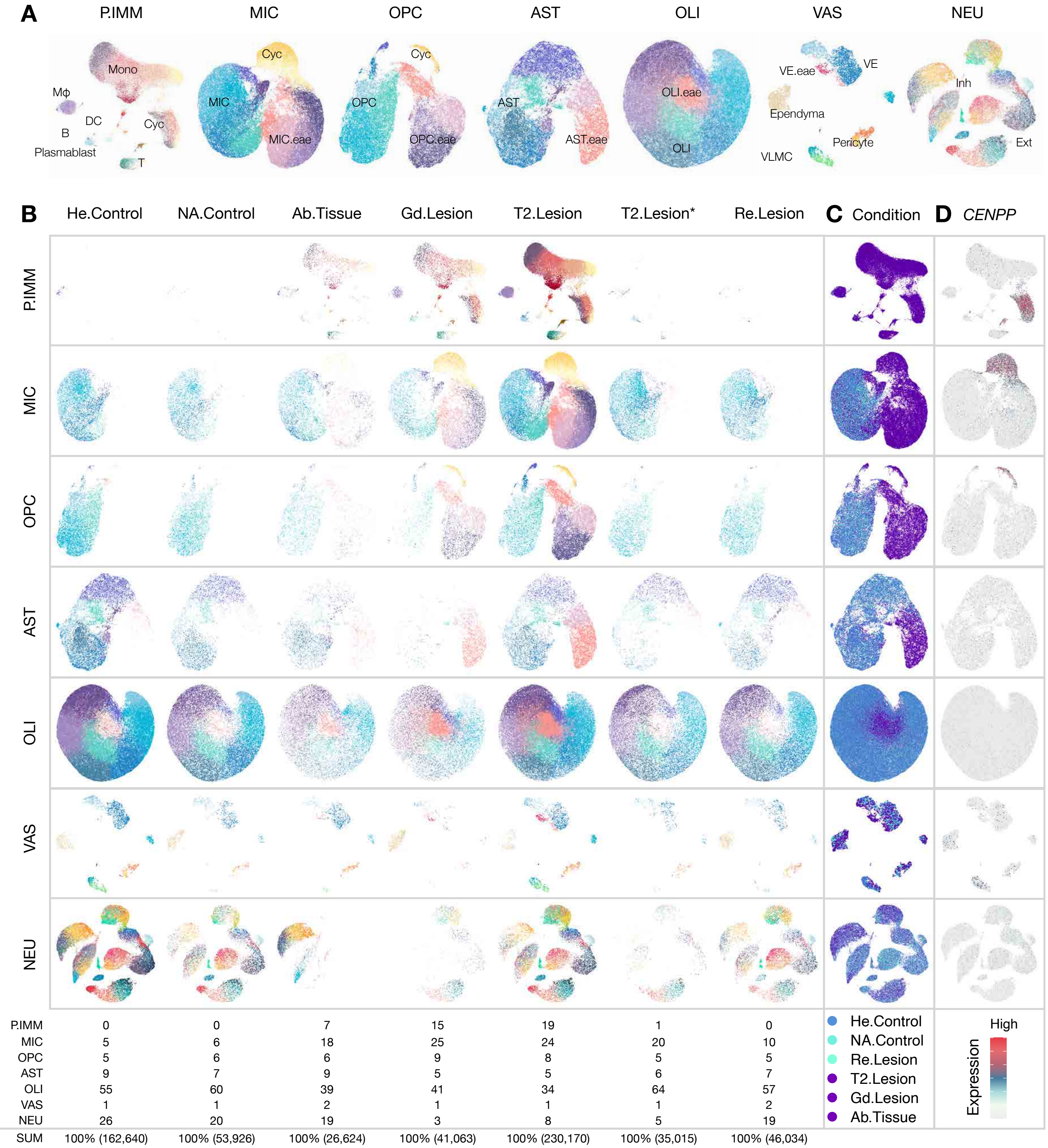

	He.Control	NA.Control	Ab.Tissue	Gd.Lesion	T2.Lesion	T2.Lesion*	Re.Lesion
P.IMM	0	0	7	15	19	1	0
MIC	5	6	18	25	24	20	10
OPC	5	6	6	9	8	5	5
AST	9	7	9	5	5	6	7
OLI	55	60	39	41	34	64	57
VAS	1	1	2	1	1	1	2
NEU	26	20	19	3	8	5	19
SUM	100% (162,640)	100% (53,926)	100% (26,624)	100% (41,063)	100% (230,170)	100% (35,015)	100% (46,034)

bioRxiv preprint doi: https://doi.org/10.1101/2023.09.25.559371; this version posted September 27, 2023. The copyright holder for this preprint (which was not certified by peer review) is the author/funder. This article is a US Government work. It is not subject to copyright under 17 USC 105 and is also made available for use under a CC0 license.

FigS10. related to Fig3. Glial and immune cells diversity across pathological states.

(A) UMAP plots of the level 2 (L2) partitions labeled with major subtypes enriched in animals with experimental autoimmune encephalomyelitis (EAE). The abbreviations: mono (monocytes), Mϕ (macrophages), DC (dendritic cells), B (B cells), T (T cells), Cyc (cycling cells), VE (vascular endothelial cells), VLMC (vascular leptomeningeal cells), Inh (inhibitory neurons), and Ext (excitatory neurons).

(B) UMAP plots split by disease conditions and colored by level 2 subclusters. This visual representation allows for the comparison and observation of cell distribution patterns specific to each disease condition. The abbreviations: He (healthy), NA (normal-appearing), Re (resolved), T2 (transverse relaxation time), Gd (gadolinium), and Ab (abnormal).

(C) UMAP plots across the L2 cell classes, colored by disease conditions. This representation helps in visualizing the dominant subclusters across different disease conditions.

(D) UMAP plots across the L2 cell classes, colored by the expression level of the CENPP (Centromere Protein P) gene. This annotation allows for the identification of cycling cells within the cell classes.

bioRxiv preprint doi: https://doi.org/10.1101/2023.09.25.559371; this version posted September 27, 2023. The copyright holder for this preprint (which was not certified by peer review) is the author/funder. This article is a US Government work. It is not subject to copyright under 17 USC 105 and is also made available for use under a CC0 license.

Fig S11 - related to Fig3

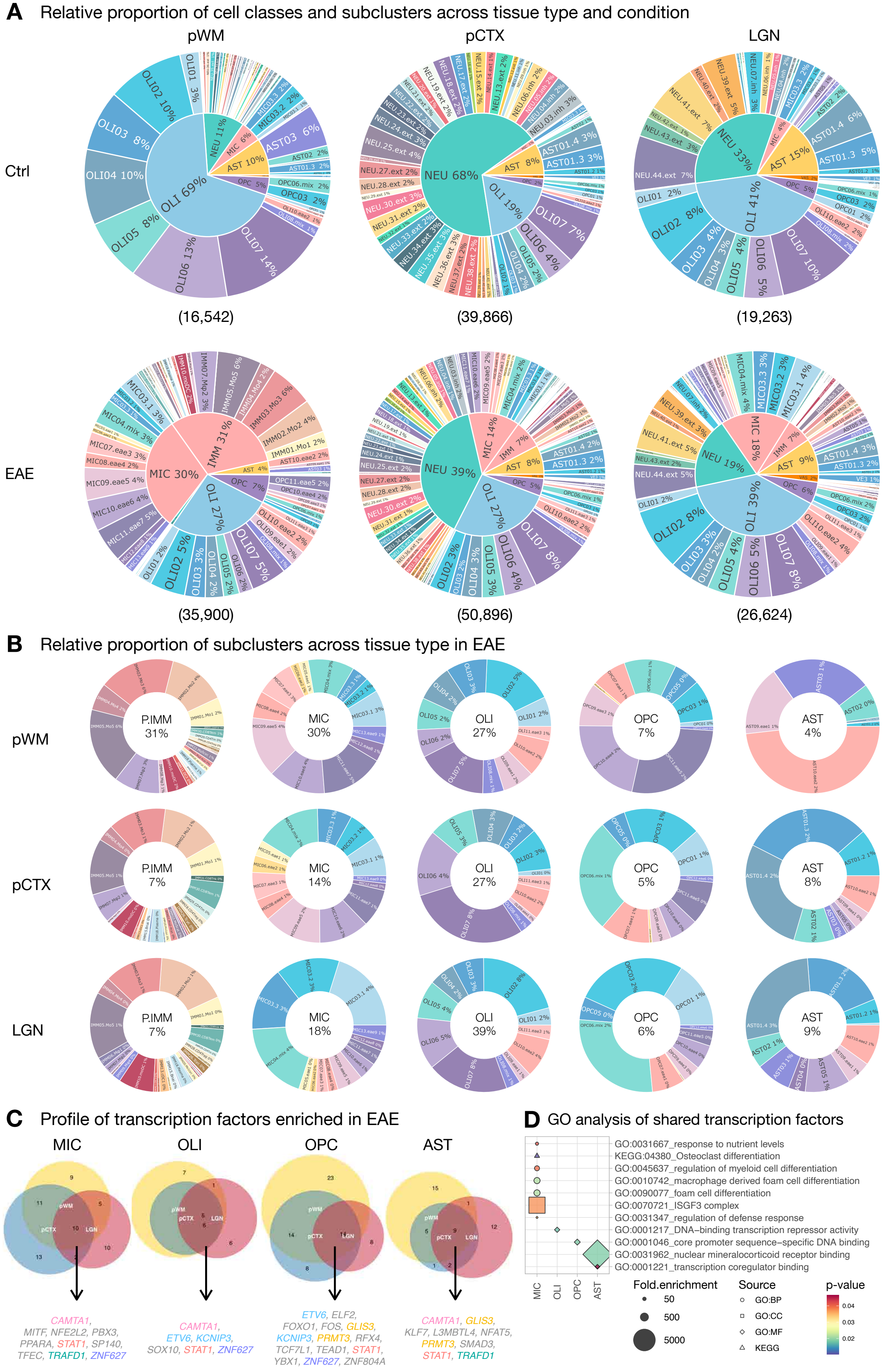

bioRxiv preprint doi: https://doi.org/10.1101/2023.09.25.559371; this version posted September 27, 2023. The copyright holder for this preprint (which was not certified by peer review) is the author/funder. This article is a US Government work. It is not subject to copyright under 17 USC 105 and is also made available for use under a CC0 license.

FigS11. related to Fig3. Comparative analysis of cell types and changes in transcription factors across different tissue types in response to EAE.

(A) The inner pie charts show the relative nuclei proportion of L1 cell classes, and the outer donut charts display L2 sub-clusters. Both control and EAE samples for each tissue type are included, with the total number of nuclei listed in parentheses. In control animals, the composition of L1 cell classes varied across tissue types, as expected. Specifically, a higher number of glial cells were found in parietal white matter (pWM), while neurons were predominant in parietal cortex (pCTX) and lateral geniculate nucleus (LGN) region. In EAE animals, there was a significant expansion of microglia (MIC) and peripheral immune cells (P.IMM) partitions in all tissue types.

(B) Donut charts show the relative nuclei proportion of glial and immune clusters in EAE animals across different tissue types. The compositions of the P.IMM and oligodendrocytes (OLI) partitions were largely similar across tissue types. However, the compositions of MIC, oligodendrocyte precursor cell (OPC), and astrocyte (AST) partitions were unique to certain tissue types. Specifically, the OPC and AST compositions were more similar in pCTX and LGN compared to pWM. On the other hand, the MIC composition was more similar in pWM and pCTX compared to LGN.

(C) Venn diagrams illustrate the similarity and diversity of transcription factors that are significantly enriched in EAE compared to control animals across different tissue types for each glial cell class. The elevated transcription factors shared by all tissue types in EAE animals are listed for each cell class, and the shared transcription factors across different cell classes are color-coded accordingly.

(D) Dot plot shows selective GO terms enriched for each list of shared transcription factors per glial cell class listed in (C).

bioRxiv preprint doi: https://doi.org/10.1101/2023.09.25.559371; this version posted September 27, 2023. The copyright holder for this preprint (which was not certified by peer review) is the author/funder. This article is a US Government work. It is not subject to copyright under 17 USC 105 and is also made available for use under a CC0 license.

Fig S12 - related to Fig3

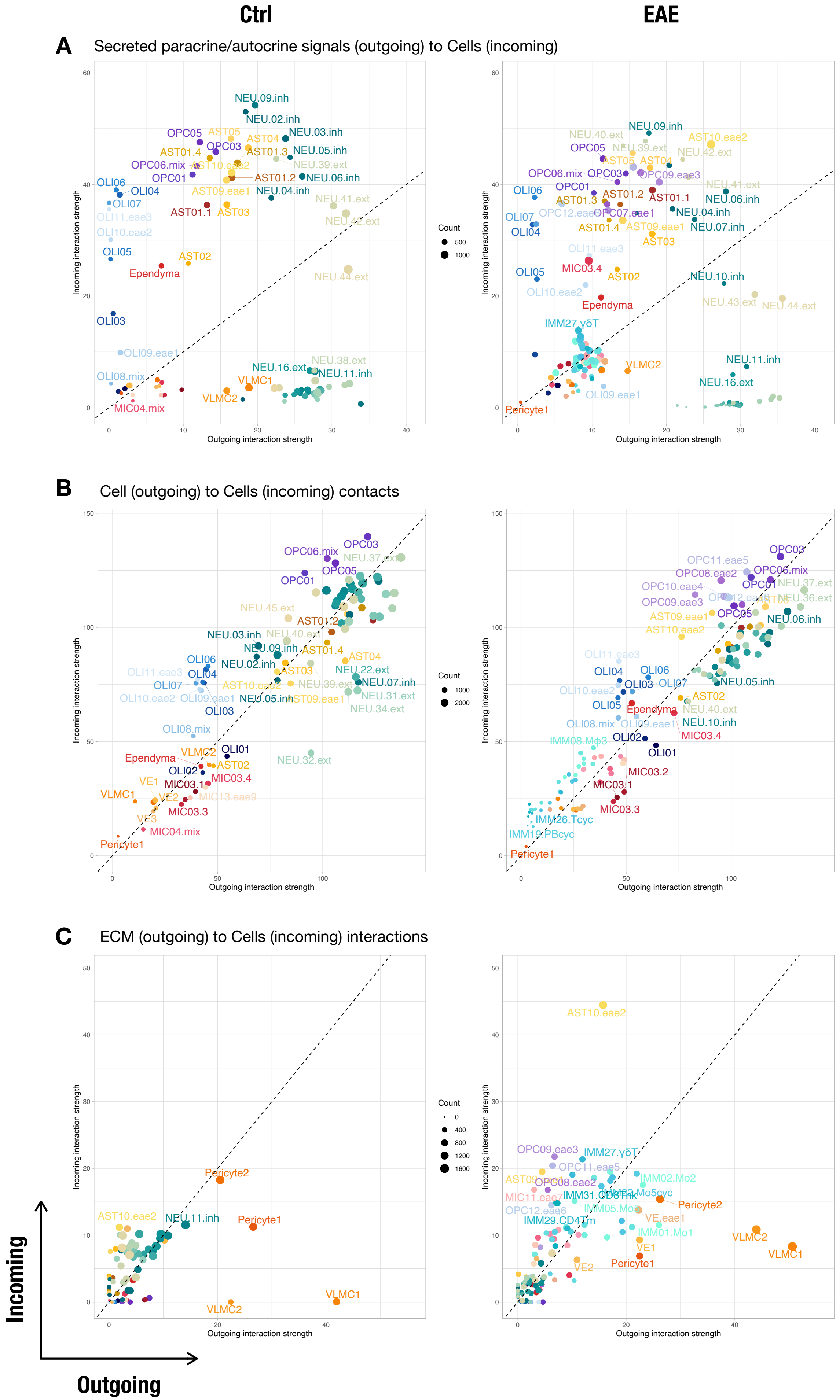

bioRxiv preprint doi: https://doi.org/10.1101/2023.09.25.559371; this version posted September 27, 2023. The copyright holder for this preprint (which was not certified by peer review) is the author/funder. This article is a US Government work. It is not subject to copyright under 17 USC 105 and is also made available for use under a CC0 license.

FigS12. related to Fig3. Communication preferences of cells within the white matter (WM) inferred by CellChat.

(A) Incoming and outgoing secreted signal-to-cell strength across different conditions.
(B) Incoming and outgoing cell-to-cell signaling strength across different conditions.
(C) Incoming and outgoing extracellular matrix (ECM) signal-to-cell strength across different conditions.

bioRxiv preprint doi: https://doi.org/10.1101/2023.09.25.559371; this version posted September 27, 2023. The copyright holder for this preprint (which was not certified by peer review) is the author/funder. This article is a US Government work. It is not subject to copyright under 17 USC 105 and is also made available for use under a CC0 license.

Fig S13 - related to Fig4

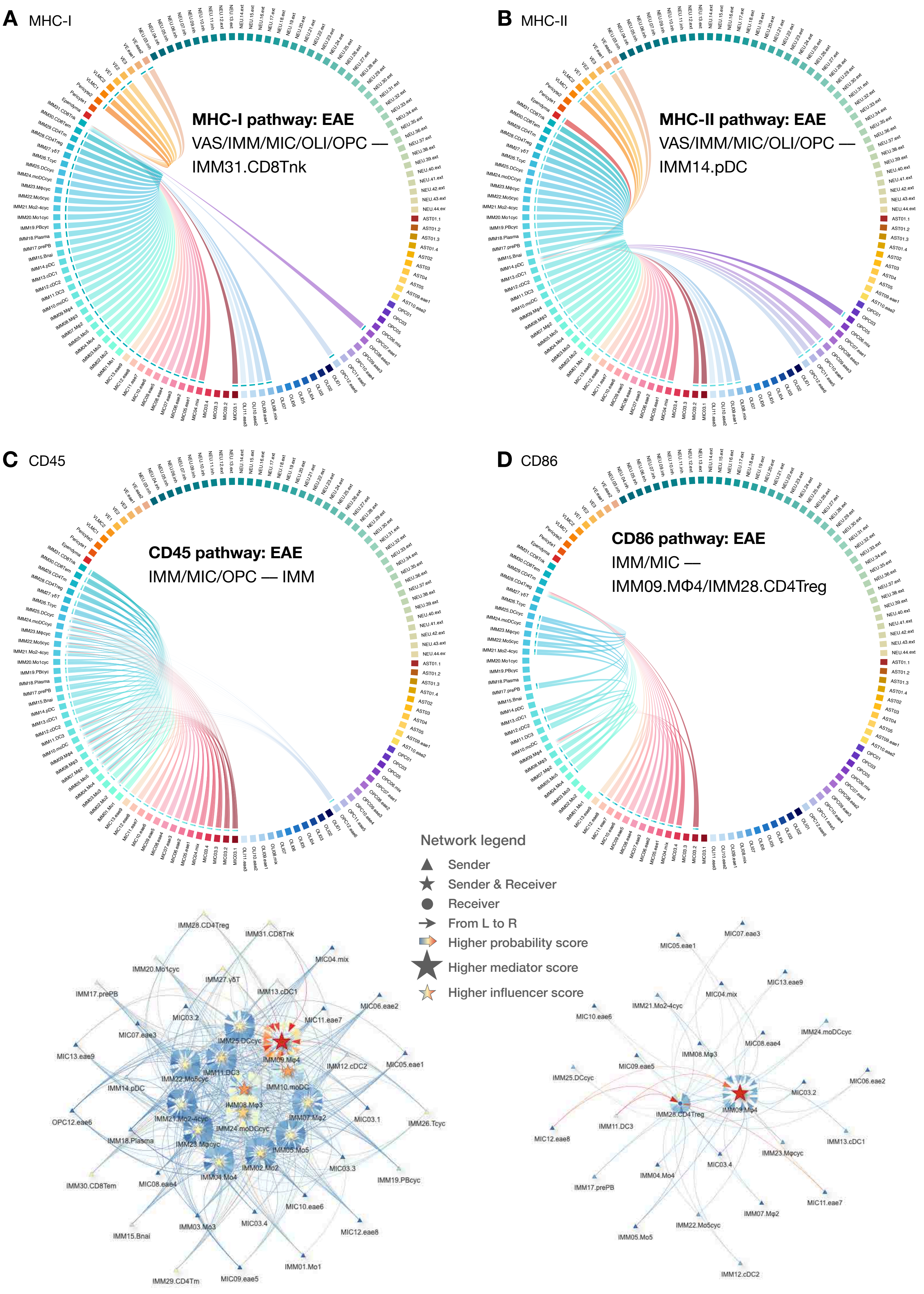

bioRxiv preprint doi: https://doi.org/10.1101/2023.09.25.559371; this version posted September 27, 2023. The copyright holder for this preprint (which was not certified by peer review) is the author/funder. This article is a US Government work. It is not subject to copyright under 17 USC 105 and is also made available for use under a CC0 license.

Fig S13.continued - related to Fig4

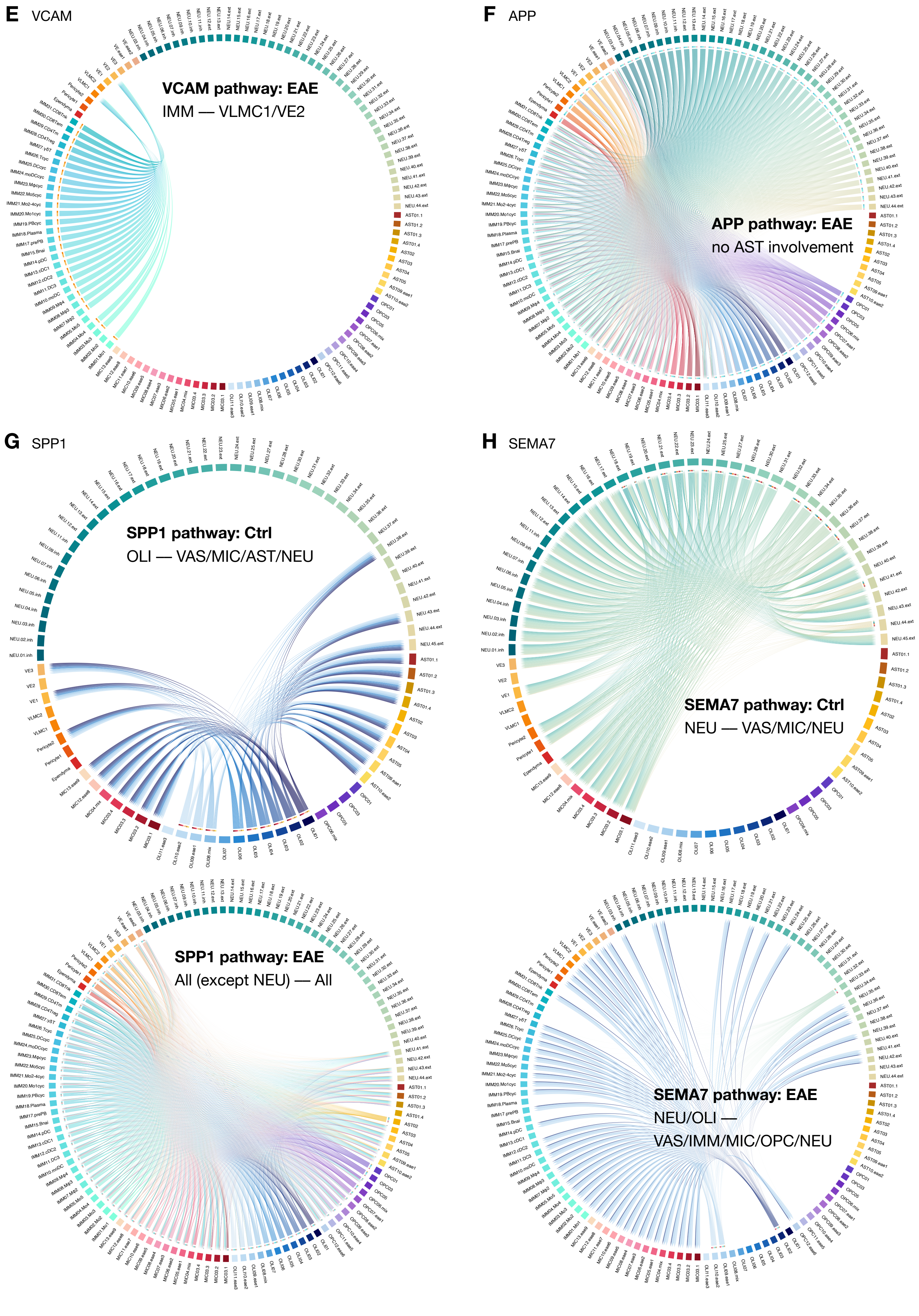

bioRxiv preprint doi: https://doi.org/10.1101/2023.09.25.559371; this version posted September 27, 2023. The copyright holder for this preprint (which was not certified by peer review) is the author/funder. This article is a US Government work. It is not subject to copyright under 17 USC 105 and is also made available for use under a CC0 license.

Fig S13.continued - related to Fig4

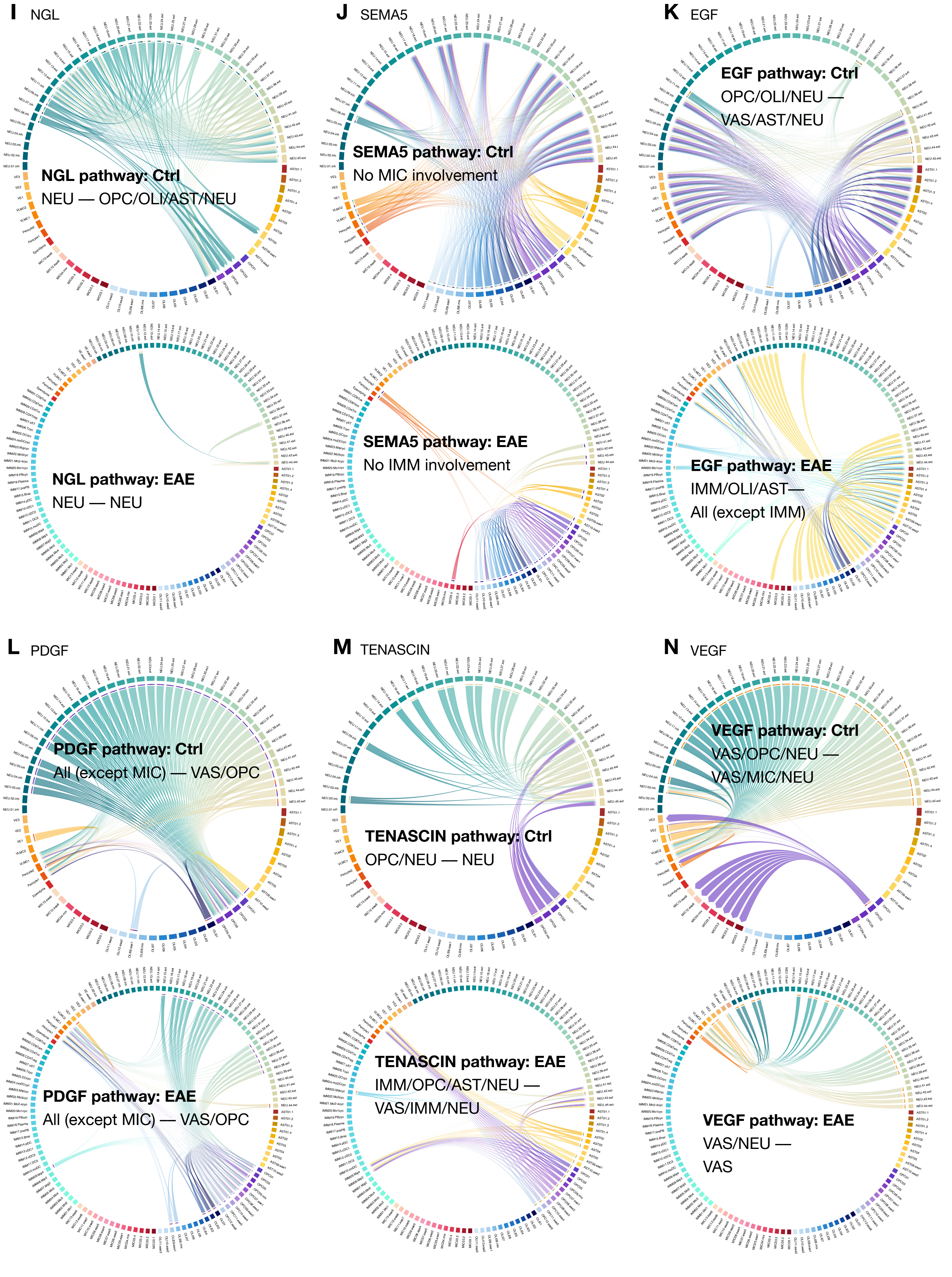

bioRxiv preprint doi: https://doi.org/10.1101/2023.09.25.559371; this version posted September 27, 2023. The copyright holder for this preprint (which was not certified by peer review) is the author/funder. This article is a US Government work. It is not subject to copyright under 17 USC 105 and is also made available for use under a CC0 license.

FigS13. related to Fig4. Chord diagrams and network plots summarize the intercellular communication profile of selected pathways.

(A) Inferred sender (ligand) and receiver (receptor) pairs of the MHC-I pathway between L2 subclusters of white matter (WM) in EAE animals.
(B) Same as (A) for MHC-II pathway in EAE animals.
(C) Same as (A) for CD45 pathway in EAE animals.
(D) Same as (A) for CD86 pathway in EAE animals.
(E) Same as (A) for VCAM pathway in EAE animals.
(F) Same as (A) for APP pathway in EAE animals.
(G) Same as (A) for SPP1 pathway in control and EAE animals.
(H) Same as (A) for SEMA7 pathway in control and EAE animals.
(I) Same as (A) for NGL pathway in control and EAE animals.
(J) Same as (A) for SEMA5 pathway in control and EAE animals.
(K) Same as (A) for EGF pathway in control and EAE animals.
(L) Same as (A) for PDGF pathway in control and EAE animals.
(M) Same as (A) for TENASCIN pathway in control and EAE animals.
(N) Same as (A) for VEGF pathway in control and EAE animals.

bioRxiv preprint doi: https://doi.org/10.1101/2023.09.25.559371; this version posted September 27, 2023. The copyright holder for this preprint (which was not certified by peer review) is the author/funder. This article is a US Government work. It is not subject to copyright under 17 USC 105 and is also made available for use under a CC0 license.

Fig S14 - related to Fig4

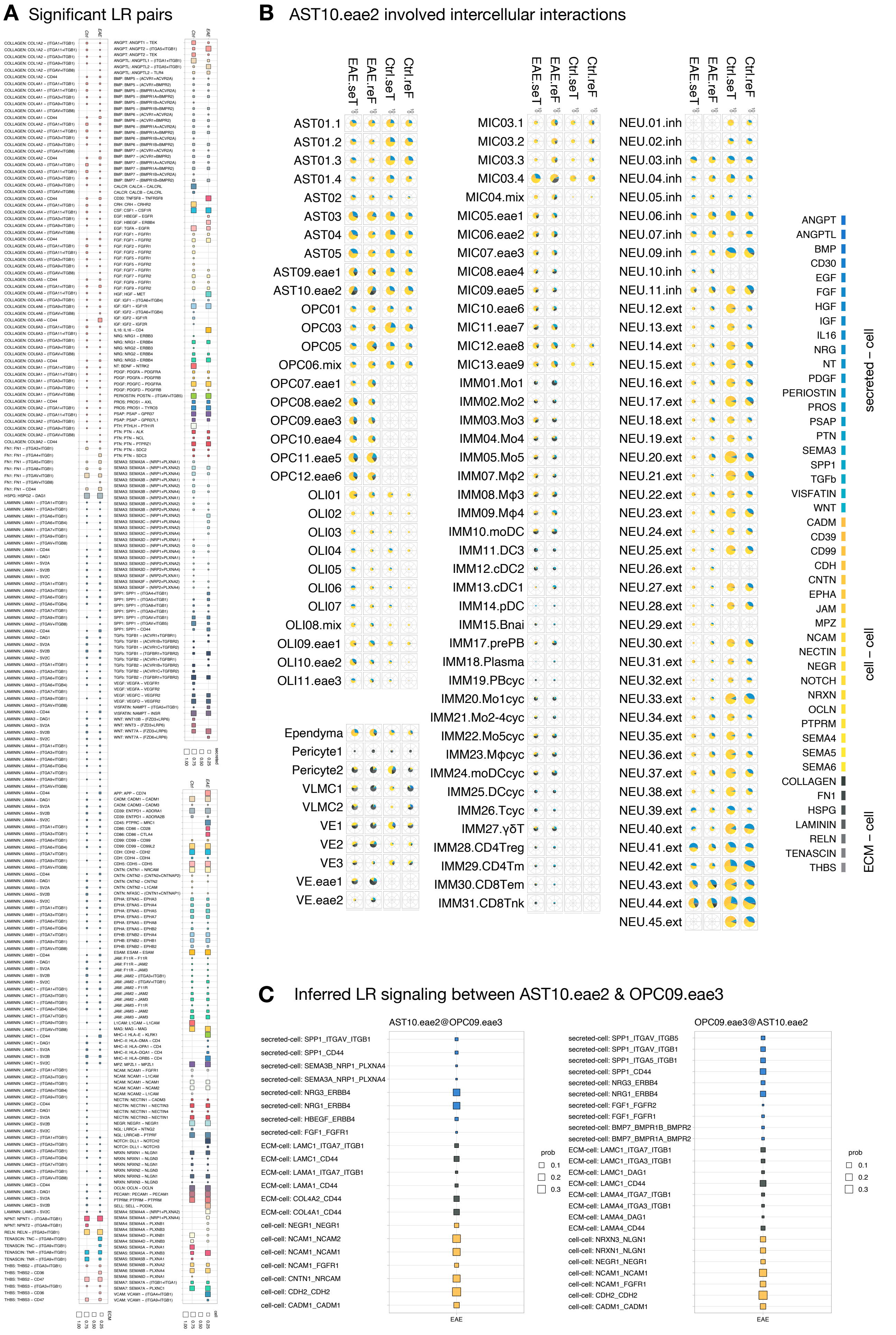

bioRxiv preprint doi: https://doi.org/10.1101/2023.09.25.559371; this version posted September 27, 2023. The copyright holder for this preprint (which was not certified by peer review) is the author/funder. This article is a US Government work. It is not subject to copyright under 17 USC 105 and is also made available for use under a CC0 license.

FigS14. related to Fig4. Significant ligand-receptor pairs inferred by CellChat across different pathological states.

(A) Dot plots showcase the relative contribution of each ligand-receptor (LR) pair to each signaling pathway; signaling strength is indicated by dot color. Interactions are categorized into 3 types: secreted autocrine/paracrine signaling interactions (secreted-cell), cell-cell contact interactions (cell-cell), and extracellular matrix (ECM)-receptor interactions (ECM-cell). Each label follows the format of "pathway name: ligand name – receptor name.”

(B) Pie charts depict the relative contribution of each pathway to overall interactions involving the AST10.eae2 subcluster per pathological state.

(C) Dot plots show the relative contribution of each LR pair to each signaling category between the AST10.eae2 and OPC09.eae3 subclusters. Each label follows the format of "pathway category: ligand name_receptor name.”
